# Adhesive Performance of Ion-Releasing Materials on Dentin with Altered Mineralization

**DOI:** 10.3390/jfb17090440

**Published:** 2026-09-01

**Authors:** Zeynep Batu Eken, Nicoleta Ilie

**Affiliations:** Department of Conservative Dentistry, Periodontology and Digital Dentistry, LMU University Hospital, LMU Medizin, LMU Munich, 80336 Munich, Germany; zeynepbatueken@gmail.com

**Keywords:** ion-releasing resin-based composite, resin-modified glass ionomer cement, glass ionomer cement, dentin adhesion, demineralization, hypermineralization

## Abstract

This study aimed to evaluate the ability of different categories of direct restorative materials to withstand artificial dentin alterations. A total of 360 human dentin specimens were allocated into 18 groups (*n* = 20). An ion-releasing bulk-fill resin-based composite (Cention Forte/CF), a resin-modified glass ionomer cement (Fuji II LC/FJLC), and an experimental conventional glass ionomer cement (EXP) were applied on sound, artificially hypermineralized, and demineralized dentin. Shear bond strength (SBS) was performed after 1 week and 6 months of storage, followed by fractographic analysis. Statistical analysis was performed using one- and three-way ANOVA, Games–Howell post hoc test, independent *t*-tests (α = 0.05), and Weibull analysis. SBS and bond reliability of CF on sound and hypermineralized dentin were higher than on other materials after both aging periods. On these two substrates, the SBS values of FJLC were higher than those of EXP, except for sound dentin after 6 months. Demineralized dentin significantly reduced the SBS of all materials under both aging conditions, with a high number of pre-test failures. The impact of aging was significant only in demineralized dentin for all materials. Ion-releasing resin-based composite performed best in terms of bond strength and reliability on sound and hypermineralized dentin. Artificially demineralized dentin severely compromised the bonding performance and maturation of all materials.

## 1. Introduction

The ultimate goal of restorative dentistry is to achieve durable adhesion between the tooth structure and restorative materials while maintaining the integrity of the healthy tooth structure. Unlike most laboratory studies that focus on bonding to sound dentin, clinicians encounter a variety of tooth substrates exhibiting structural differences in routine practice, particularly carious lesions. Management of such lesions involves a minimally invasive approach to help preserve healthy tissue through selective caries removal. According to this concept, caries-affected dentin should be retained following the removal of the heavily and irreversibly degraded caries-infected dentin that contains bacteria [1]. Caries-affected dentin exhibits a lower mineral content, a higher degree of porosity, and altered collagen organization compared with sound dentin [2,3]. This partially demineralized layer retains the potential for remineralization [4].

Another frequently bonded tooth structure is sclerotic dentin, a highly mineralized tissue commonly found in non-carious cervical lesions in elderly individuals. Sclerotic dentin results from the natural defense mechanism in response to stimuli and is characterized by occluded dentinal tubules with rhombohedral, whitlockite crystallites, mineral deposition and a hypermineralized surface layer [5,6]. This unique structure leads to reduced adhesion performance [7,8]. Therefore, appropriate mechanical and chemical retention to these clinically challenging substrates should be achieved. Although resin-based composites (RBCs) are mostly the first-choice restorative material, it is not always possible to fulfill the requirements of technique-sensitive steps, especially for patients with lower compliance. Along with increased life expectancy, complex health conditions, difficulty performing oral hygiene, and limited tolerance for chair time, management of lesions in elderly patients has gained importance.

Varying restoration failure rates across age groups [9] may be due to differing levels of compliance and require treatments that can inhibit caries progression. Therefore, the current trend favors functional biomaterials—simple-to-use restorative materials with therapeutic potential to prevent secondary caries formation and support remineralization of the tooth structure [10]. Glass ionomer cements (GICs) have been commonly used for this purpose due to their bioactive, ion-releasing properties, which are closely linked to their inherently porous microstructure. They have the ability to neutralize acidic conditions and promote remineralization through ion release [11]. Resin-modified glass ionomer cements (RMGICs) are another amalgam-alternative material group that combines the advantages of GICs and RBCs. With the addition of resin and polymerization reaction, lower early-moisture sensitivity, reduced setting time, and improved mechanical properties over GICs have been provided [12,13]. However, similar to GICs, insufficient mechanical properties limit their indications in stress-bearing regions, leading to higher failure rates in the posterior region [14]. Since RBCs lack the buffering potential to increase pH, unlike materials with bioactive properties [15], there is a trend toward developing modified RBCs with caries-prevention potential. Therefore, research has focused on developing new self-adhesive and ion-releasing tooth-colored restorative materials with enhanced mechanical properties in addition to remineralization and caries-inhibition potential together with reduced technique sensitivity [16].

Such materials are offered as bulk-fill dual-cure RBCs containing bioactive glass fillers that release ions contributing to pH regulation and inhibition of demineralization [17,18]. Since the current literature indicates promising bioactive potential and comparable clinical performance to bioinert RBCs [19,20], the material is a promising alternative for the treatment of carious lesions [21]. At the same time, ion-releasing materials may have a positive impact on pediatric and geriatric patients at higher risk of developing caries.

Considering the wide range of available materials, a clear comparison and understanding of their abilities would help clinicians select the most appropriate option based on patients’ needs, the type of restoration and the clinical situation. With a view to a simplified restoration concept that can reduce treatment costs by shortening treatment time and is also suitable for patients with limited compliance, bioactive bulk-fill RBCs, RMGICs and GICs are potentially all materials of choice.

In addition, understanding how these materials interact with various altered surfaces is essential for achieving durable restorations. Due to the difficulty in standardization and reproducibility of the natural substrates, artificial laboratory models offer the advantage of simulating chemical changes, allowing for comparing the materials’ performances. pH-cycling and artificial hypermineralization protocols have been used to approximate caries-affected and sclerotic dentin, respectively. This may help clinicians understand which material category is less affected by altered dentin substrates under laboratory aging conditions. Therefore, this study aimed to evaluate and compare the bond strength, the reliability of the bond, and the fracture behavior of ion-releasing restorative materials used for a simplified restoration concept bonded to artificially hyper- and demineralized dentin versus sound dentine while involving aging. The null hypotheses were that (i) material type, (ii) dentin alteration, and (iii) aging show no impact on the bond strength and fracture mechanisms.

## 2. Materials and Methods

### 2.1. Specimen Preparation

An a priori power analysis using G*Power software (version 3.1, Heinrich-Heine-Universitat, Düsseldorf, Germany) indicated that a total sample size of 252 (*n* = 14) would be enough to detect a medium effect (f = 0.25) with a power of 90% using analysis of variance (ANOVA) between means of 18 groups with an alpha error probability of α = 0.05. In order to perform Weibull analysis, the sample size was increased to 20. A total of 97 non-carious human third molars were collected and stored in a 0.2% sodium azide solution at room temperature. The roots of the teeth were separated slightly below the cemento–enamel junction with a low-speed diamond saw (IsoMet, Buehler, Lake Bluff, IL, USA). A subsequent horizontal cut at the midcoronal level above the pulp chamber was performed to obtain coronal and cervical halves. Furthermore, the two dentin surfaces were cut vertically into pieces, resulting in 4 specimens per tooth, while, depending on the tooth size, 2 or 6 specimens were obtained. A total of 360 dentin specimens were randomly assigned to 18 groups (*n* = 20). Group composition was controlled by distributing the specimens obtained from the same tooth into different groups, with opposing surfaces allocated to separate aging groups within the same material and dentin substrate. Specimens were embedded in methacrylate resin (Technovit 4004, Kulzer, Hanau, Germany) in stainless-steel cylinders and ground flat with 600-grit silicon carbide paper (Leco, St. Joseph, MI, USA) under running water to obtain a standardized smear layer.

### 2.2. Experimental Procedure

Specimens without any alteration served as controls (sound dentin). The surfaces of the specimens allocated for hypermineralization and demineralization were covered by nail varnish, leaving only the occlusal dentin area exposed. The exposed dentin surfaces of the specimens belonging to the hypermineralization group were etched with 35% phosphoric acid for 5 s. The specimens were suspended in hypermineralization solution (1.5 mM CaCl_2_ 2H_2_O, 0.9 mM K_2_HPO_4_ and 0.15 M KCl) adjusted to pH 7.0 for 14 days [7], and the solutions were changed daily.

Specimens allocated for demineralization were subjected to pH cycling [22] for 14 days. They were suspended in demineralization solution (1.5 mM CaCl_2_, 0.9 mM KH_2_PO_4_ and 50 mM acetic acid) adjusted to pH 5.0 for 8 h, followed by immersion in remineralization solution (1.5 mM CaCl_2_, 0.9 mM NaH_2_PO_4_, 0.13 M KCl and 20 mM HEPES) adjusted to pH 7.0 for 16 h. New solutions were used for each cycle, and the specimens were rinsed with distilled water after each cycle. All the solutions were kept at room temperature and continuously stirred.

The tested restorative materials in this study were: (1) ion-releasing bulk-fill RBC [Cention Forte (CF), Ivoclar Vivadent, Schaan, Liechtenstein], (2) RMGIC [Fuji II LC Capsule (FJLC), GC Europe, Leuven, Belgium], and (3) GIC [Experimental GI Filling Material (EXP), Solventum, St. Paul, MN, USA]. Table 1 shows the chemical compositions of the tested materials. Prior to application of CF, a primer (Cention Primer, Ivoclar Vivadent, Schaan, Liechtenstein) was applied for 10 s and dispersed with air until a shiny, thin, immobile film was formed. For FJLC, a conditioner (Cavity Conditioner, GC Europe, Leuven, Belgium) was applied for 10 s, rinsed thoroughly and gently air dried. No pretreatment was applied for the EXP specimens.

Following the capsule activation, mixed restorative materials were inserted into a split mold with a diameter of 2.4 mm (Ultradent Products; South Jordan, UT, USA) under standard ambient laboratory conditions. CF and FJLC were polymerized with a violet-blue LED light curing unit (Bluephase^®^ Style, Ivoclar Vivadent; Schaan, Liechtenstein) with an irradiance of 1366.6 ± 3.8 mW/cm^2^ and a light-emitting window of 10 mm diameter above the central opening of the mold for 15 s and 20 s, respectively. A spectrophotometer (Managing Accurate Resin Curing, Bluelight Analytics Inc., Halifax, NS, Canada) was used to determine irradiance, radiant exposure, and spectral distribution. Specimens prepared with EXP and FJLC were kept in the mold in a vapor-saturated atmosphere at 37 °C for 1 h. FJLC build-ups were covered with a coating material (GC Fuji Varnish, GC Europe, Leuven, Belgium) and gently air dried. Following the removal of the molds, all specimens were stored in distilled water at 37 °C for 1 week or 6 months. The distilled water was changed regularly every two weeks until testing.

### 2.3. Shear Bond Strength (SBS) Testing

The testing was carried out with a Universal Testing Machine (Z2.5, Zwick/Roell, Ulm, Germany), adapted from ISO-29022 by replacing a notched-edge chisel with a straight-edge chisel, at a crosshead speed of 0.5 mm/min until fracture. The SBS values of each specimen were calculated by dividing the load at fracture by the individual bonding area that was measured in a light microscope after debonding. When specimens failed prior to testing, they were recorded as pre-test failure (ptf) and included in the calculation of mean values. If ptf occurred during demolding or aging before the bond strength testing, the load was recorded as 0 N. If a ptf occurred during the placement of the chisel while bond strength testing, the load of failure was assigned as 0.05 N, considering the load of the chisel.

### 2.4. Fractographic Analysis

The fractured specimens were examined with a stereomicroscope (Stemi 508, Carl Zeiss Microscopy GmbH, Göttingen, Germany) following the completion of the SBS test. Both sides of the fractured specimens—the dentin surface and the restorative material surface—were evaluated with a camera extension (Axiocam color 305, Carl Zeiss Microscopy GmbH, Göttingen, Germany) and AxioVision 4.9.1 computer software. The modes of fracture were distinguished in “adhesive” failure when a fracture line was located between the tooth and restorative material and “cohesive” failure when the fracture line proceeded either through dentin or material and involved a bulk fracture. Adhesive failures were further classified based on the coverage of a thin layer of material remnants on the dentin surface as follows: approximately less than 25%, between 25 and 75%, and more than 75%.

### 2.5. Scanning Electron Microscopy (SEM) Evaluation

Two additional specimens were prepared to observe the effects of the hyper- and demineralization protocols on the surface ultrastructure. Teeth were horizontally cut at the midcoronal level above the pulp chamber, then ground flat with 600-grit silicon carbide paper (Leco, St. Joseph, MI, USA) under running water. The resulting specimens were allocated into either the hyper- or demineralization protocol. Following the completion of the surface-altering protocols, the specimens were successively dehydrated with increasing concentrations of ethanol solutions (25%—20 min; 50%—20 min; 75%—20 min; 95%—30 min; ≥99.8%—60 min) and sputter-coated with gold. SEM analysis (Zeiss Supra 55 V P, Carl Zeiss AG, Oberkochen, Germany) was performed at a beam voltage of 10 kV.

Subsequently, the validity of the artificial demineralization method was further verified by conducting elemental analysis prior to the experimental phase. Three additional demineralized dentin specimens were cross-sectioned parallel to the longitudinal axis of the tooth and sputter-coated with gold. The cross-profiles of the specimens were analyzed by SEM-energy-dispersive spectroscopy (SEM-EDX) connected to the SEM presented above under a magnification of 126Χ. For each specimen, three line-scans of phosphorus (P) and calcium (Ca) variation were collected, starting from the demineralized surface towards the underlying sound dentin over a depth of 600 µm. The data from the three measurements of each specimen were fitted using an exponential equation with a rise to maximum; the parameters a, b and R^2^ were indicated for each specimen.

### 2.6. Statistical Analysis

The data were analyzed using SPSS (IBM SPSS Statistics, Version 29, International Business Machines Corporation, Armonk, NY, USA). The Shapiro–Wilk test was used to check for normal distribution. Equality of variances was determined with Levene’s test. A three-way ANOVA was employed to evaluate the influence of each variable. Higher values of the partial eta-squared (ηp^2^) indicate a larger effect on the measured data. One-way ANOVA with a Games–Howell post hoc test was used for further comparison of the parameters. Independent-samples *t*-tests were used to evaluate the influence of aging. The results were considered statistically significant for *p* < 0.05. Furthermore, Weibull statistics were applied to evaluate the reliability of each material under the tested substrate and aging conditions.

## 3. Results

The Shapiro–Wilk test confirmed a normal distribution for 14 out of 18 groups. Data were therefore considered normally distributed. As some SBS data revealed inequality of variances, mean values were compared by ANOVA with Games–Howell post hoc tests. The SBS and Weibull statistics for each group are presented in Table 2.

Three-way ANOVA revealed highly significant influences of the parameters “material” (*p* < 0.001, ηp^2^ = 0.642) and “substrate” (*p* < 0.001, ηp^2^ = 0.607), while the impact of “aging” was lower (*p* = 0.002, ηp^2^ = 0.028). The binary combination of the parameters “material and substrate” (*p* < 0.001, ηp^2^ = 0.412) also displayed a significant influence on SBS. The tooth had no significant influence on the parameters investigated (*p* > 0.05).

The highest SBS was achieved with CF in the sound and hypermineralized dentin groups under both aging conditions (*p* < 0.001). The results identified higher SBS for FJLC than EXP on these two substrates, except for sound dentin after 6 months (*p* = 0.123). Hypermineralization significantly increased the SBS of CF after 1 week compared to sound dentin (*p* = 0.008), while no statistically significant differences were observed for other materials. Demineralized dentin significantly reduced the SBS values of all materials under both aging conditions. When comparing the materials on demineralized dentin after 1 week of aging, CF showed the highest, while FJLC showed the lowest SBS (*p* < 0.001). After 6 months, all specimens of FJLC failed before testing, and there was no significant difference between CF and EXP (*p* = 0.147). A statistically significant decrease in SBS by aging was observed with independent *t*-tests for all materials bonded to demineralized dentin (*p* < 0.001), along with higher ptf numbers in the 6-month groups (CF: 2, FJLC: 20, EXP: 8). The percentage reduction values of CF, FJLC and EXP by time were 90.6%, 100% and 88%, respectively. The impact of aging was not significant in the sound and hypermineralized dentin groups.

**Figure 1:**
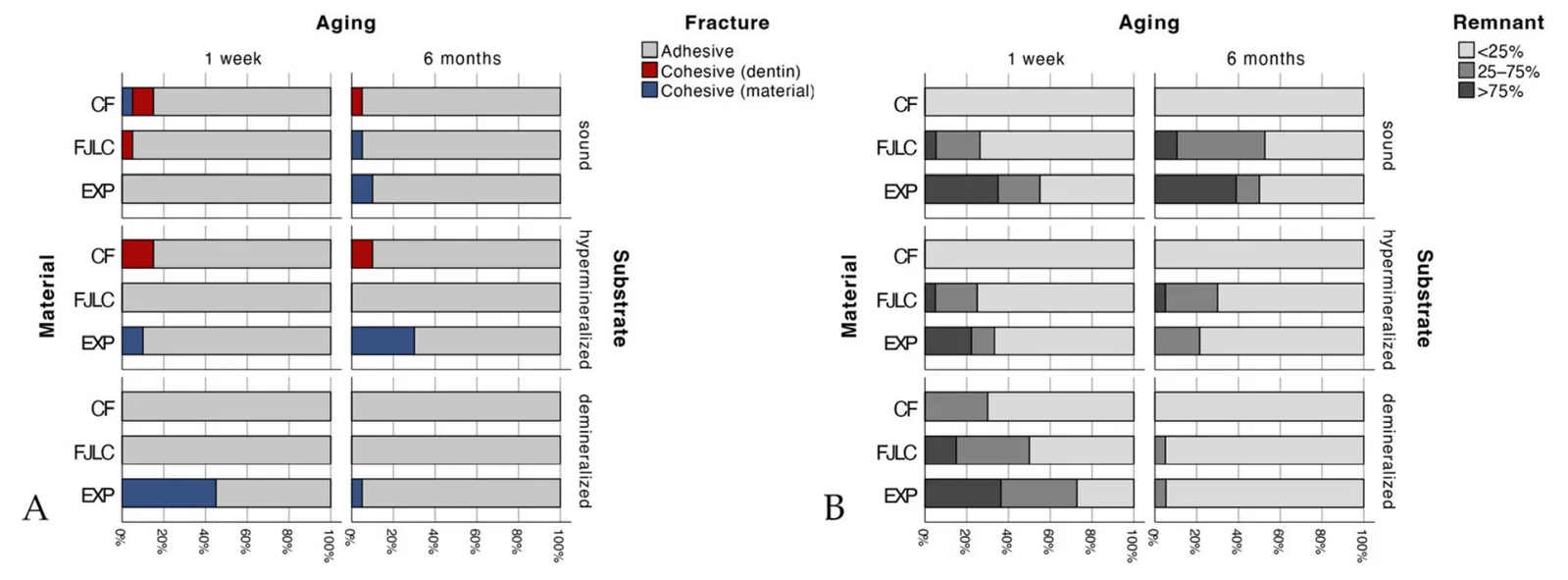
(**A**) Fracture failure modes in percent (%) and (**B**) remnant in percent for the adhesively fractured specimens.

**Figure 2:**
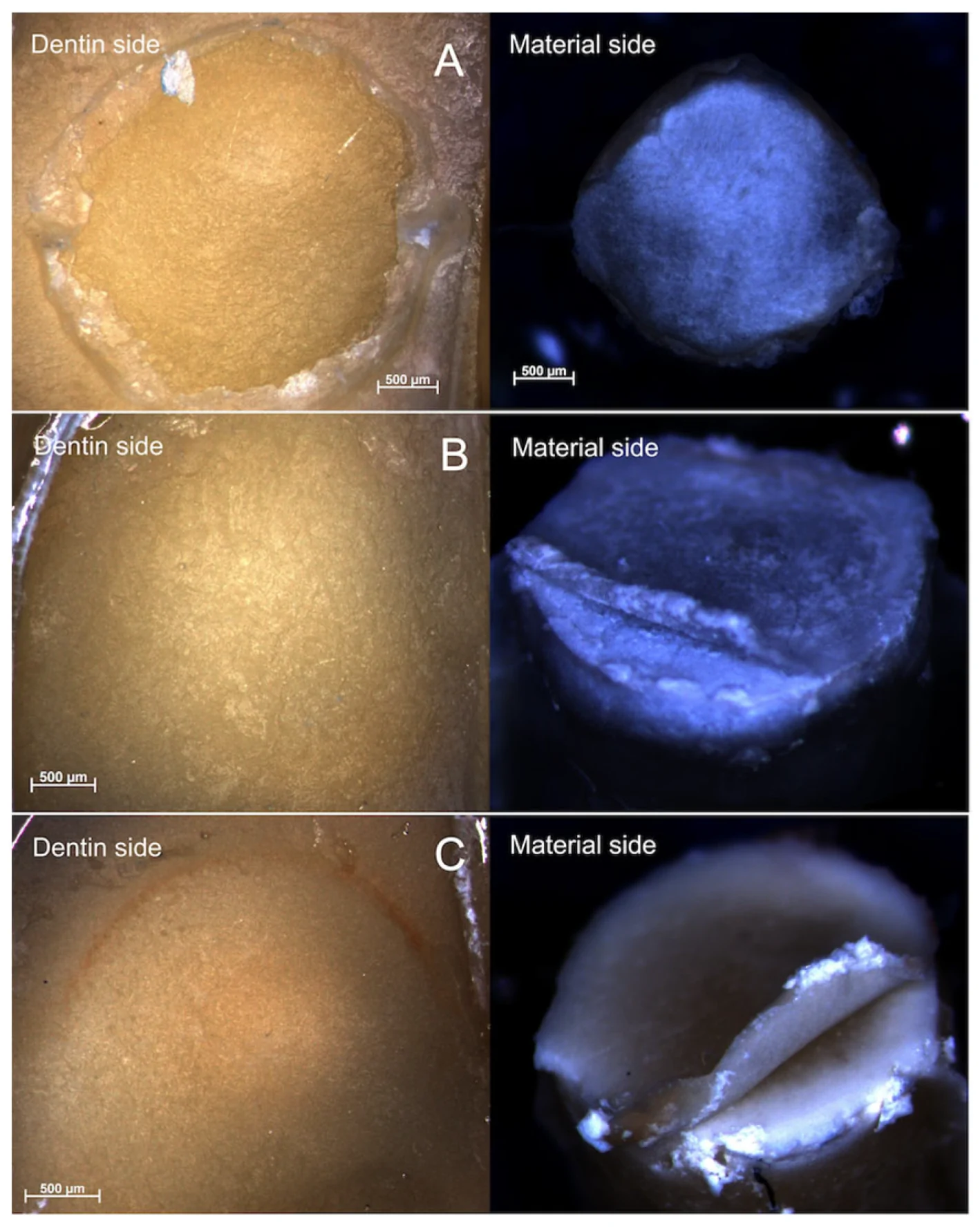
Representative images of the fractured specimens CF (**A**), FJLC (**B**) and EXP (**C**) on demineralized dentin after 6 months of aging. A unique layer specific to this substrate was observed between the materials and the surface, which tended to shrink and detach from the material surface as it dried out.

The distribution of failure modes is shown in Figure 1. Specimens predominantly failed adhesively. Few cohesive failures in dentin were recorded, mainly for CF (6.7% of all CF specimens), while cohesive failures in material were higher for EXP (16.7% of all EXP specimens). Cohesive failures of EXP were observed in the 1-week demineralized dentin group (45%) and mostly appeared as fractures at the edges of the specimens. All ptfs were interfacial failures. When the adhesively failed specimens were further observed, a thin layer of material remnant was recorded more often on FJLC and EXP than on CF. This thin layer was considered the interaction layer if there was no bulk fracture of material that increased the surface area meaningfully. The morphology of the interface differed visibly on demineralized dentin. A unique layer between the material and demineralized dentin was observed that became more visible after 6 months of aging. Representative images were selected based on the evaluation of all specimens, reflecting the observed morphological pattern within that group (Figure 2). Debonding mostly occurred between the demineralized dentin and this layer following 6 months of maturation. It was observed that the layer tended to collapse and separate from the material surface as it dried out.

Further analysis of the SBS results was employed for the only adhesively failed specimens (91.4%). Excluding cohesive failures resulted in a slight reduction that influenced the material comparisons of demineralized dentin. After 1 week, the difference between FJLC and EXP became insignificant (FJLC = 0.8 ± 0.8 MPa, EXP = 2.1 ± 1.7 MPa; *p* = 0.098), whereas after 6 months, the difference between CF and EXP on the demineralized dentin became significant (CF = 0.5 ± 0.3 MPa, EXP = 0.2 ± 0.3 MPa; *p* = 0.021).

**Figure 3:**
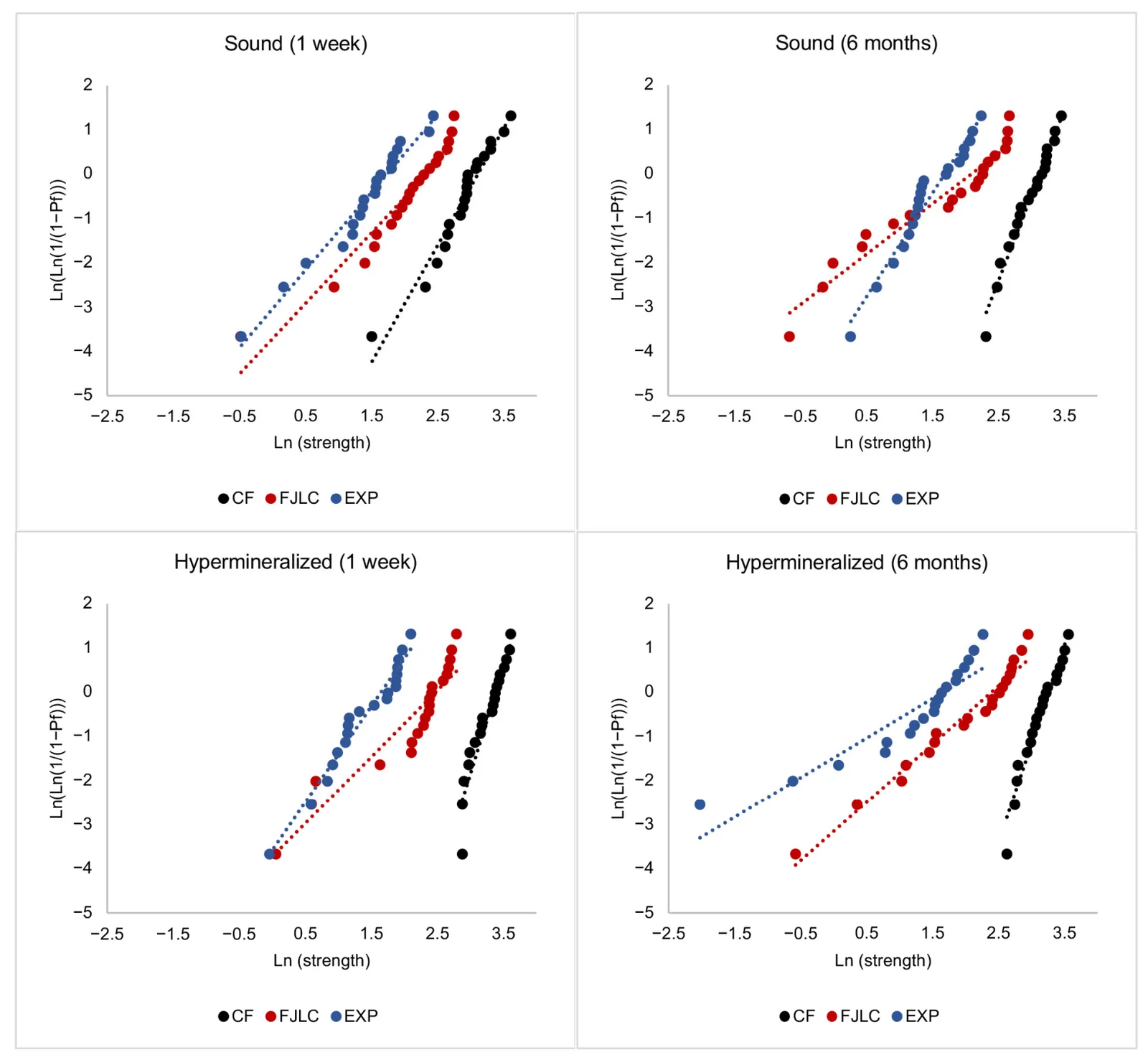
Weibull distribution as a function of material and substrate for both aging conditions; Pf = probability of failure.

The Weibull distribution is illustrated in Figure 3, and the corresponding Weibull statistics of the SBS data are presented in Table 2. Differences between the Weibull moduli (m) were considered significant when the 95% confidence intervals did not intersect. Ptfs were not included in the Weibull analysis. Due to the high number of ptfs, Weibull statistics were not applied to demineralized dentin groups. Both after 1 week and 6 months, CF had higher m values than FJLC and EXP on both substrates.

Hypermineralization enhanced the reliability of CF and EXP after 1 week compared to the sound dentin. Following aging, m of CF on hypermineralized dentin was equal to that of sound, whereas EXP reduced significantly. FJLC remained unaffected by the substrate at both storage periods. Aging had a positive impact on the Weibull modulus of CF and EXP on sound dentin, whereas a slightly negative effect was observed on FJLC. On hypermineralized dentin, the reliability of EXP was reduced by aging, while it remained stable for CF and FJLC.

**Figure 4:**
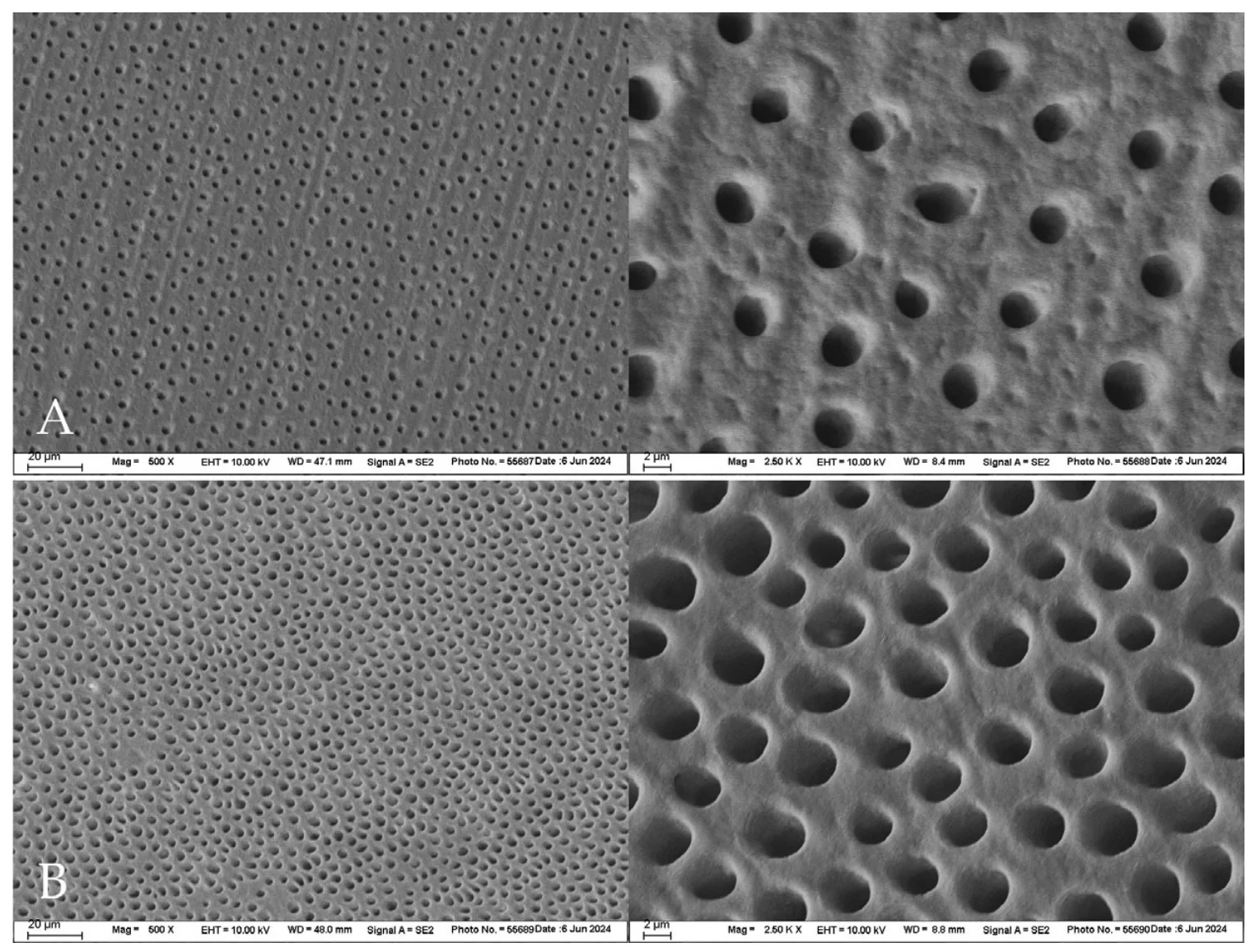
SEM images of hypermineralized (**A**) and demineralized (**B**) dentin surfaces at 500× (**left**) and 2500× magnification (**right**).

**Figure 5:**
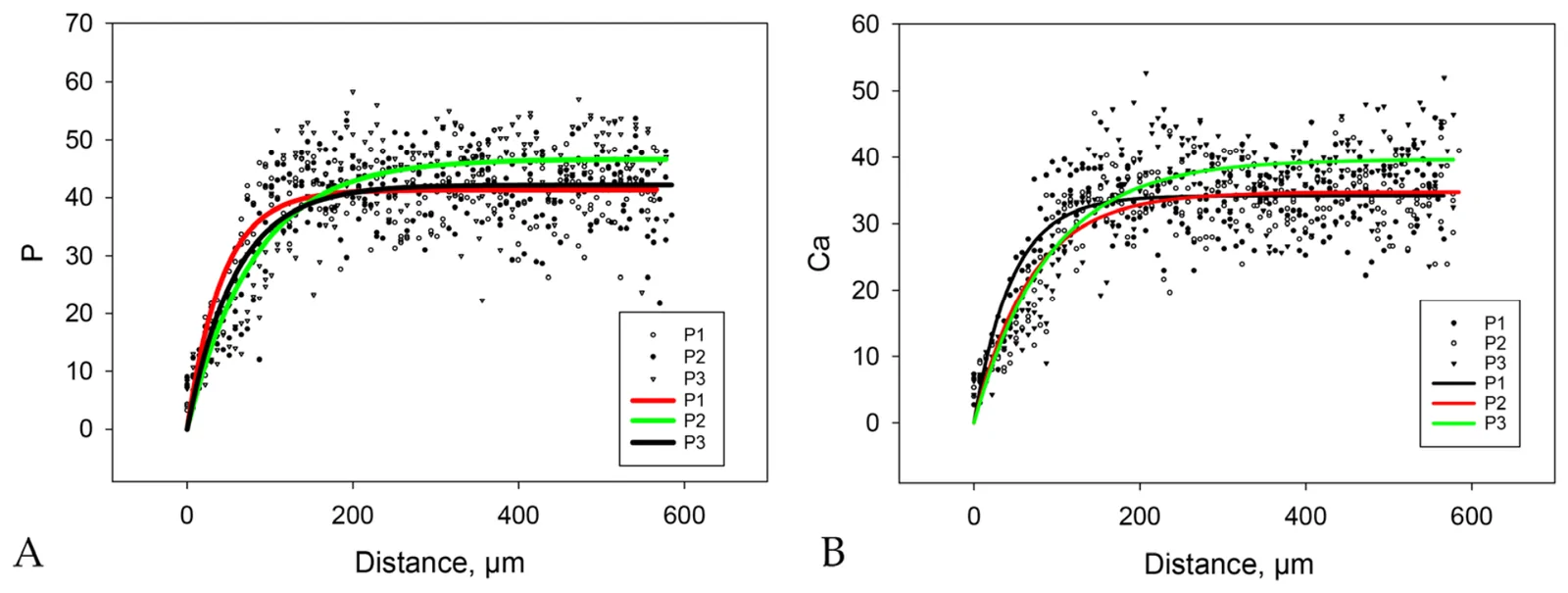
EDX line-scan depth profiles of P (**A**) and Ca (**B**) with exponential model fitting of three demineralized dentin specimens. Three profiles were performed in each specimen.

The SEM images in Figure 4 show the dentin surfaces after artificial hypermineralization and demineralization. Hypermineralization revealed narrow dentinal tubules, while a higher number of tubules with enlarged diameters were observed with the pH-cycling demineralization method. EDX line scan analysis of the cross-section of demineralized dentin surfaces identified the gradual increase in mineral content from the lesion surface followed by a plateau. The fitted model parameters (a and b) and R^2^ values for each specimen are presented in Table 3. The corresponding fitted curves for both P and Ca are shown in Figure 5, indicating an average demineralization depth of approximately 100 µm.

The demineralization of dentin over depth, starting in the sound dentin at a depth of 600 µm from the demineralized surface, was mathematically fitted using an exponential function:

$$y=a*(1-e^{-bx})$$

where the parameters *a* and *b* are modulation factors of the exponential function.

## 4. Discussion

Altered substrates with different levels of mineralization could affect the adhesion of restorative materials in clinical practice [5,23]. To address these clinically challenging substrates and conditions, particularly with regard to finding suitable alternatives to amalgam, simplified and cost-effective restorative materials and methods are being sought that allow for rapid application with reduced technique sensitivity. The study therefore focuses on materials that can be used in bulk, i.e., in a thick single layer, and also have the ability to exhibit caries-protective and/or remineralizing effects. Dual-cured bulk-fill RBCs with bioactive fillers, GICs and RMGICs met these requirements and were therefore compared in view of their bonding effectiveness and durability on sound and artificially altered dentin substrates.

The superior performance of RBCs compared to GICs under ideal conditions has been a well-known fact [14]. For the most recently released bulk-fill RBC with bioactive filler (CF), limited information is available regarding its bonding performance. The predecessor of CF, Cention N, was formulated as a self-adhesive material, requiring no additional pretreatment. A clear improvement in dentin bond strength was observed when it was used in conjunction with a bonding agent [24,25]. In such cases, bond strength exceeds that of GIC and RMGIC [24,26], yet this benefit is lost in the absence of pretreatment [24,25]. As a subsequent development, CF has been recommended for use with a dedicated primer that does not need light curing. FJLC was selected as the representative material for the RMGIC material category due to its widespread use in the previous literature and was used along with a polyacrylic acid conditioner, which is known to enhance its bond strength [27]. In contrast, EXP is an experimental GIC with promising enhanced mechanical properties (data not yet published) that can be used without conditioning agents or coatings. Irrespective of the substrate alteration or aging of the bond, bond strength decreases in the material sequence CF > FJLC > EXP. Consistent with our results, CF was shown to perform better compared to RMGIC and GIC in the recent literature [28,29,30,31]. Regarding the comparison of RMGICs to conventional GICs, various previous studies showed better bonding potential of FJLC than the latter [32,33]. The differing bond strengths of the three tested materials in the present study led to the rejection of the first null hypothesis.

Differences in bond strength and the behavior of the bonding during aging need to be related to the adhesion mechanism of the tooth substrate. The GICs and RMGICs involve a chemical bonding achieved by the formation of ionic bonds between the calcium ions in the hydroxyapatite of the tooth structure and the carboxyl groups of the polyalkenoic acid, along with micromechanical interlocking to dentin through infiltration into the collagen network. An ion-exchange interfacial layer has been described at the GIC–dentin interface that is formed via reciprocal diffusion of ions [34,35]. This interaction layer is considered to mature over time and may be responsible for the durability of the adhesion over time [36]. This makes the assessment of immediate and aged bonding performance of ion-releasing materials essential for the evaluation of material maturation. Accordingly, 6 months of distilled water storage was preferred in the present study over accelerated aging methods to allow real-time interface maturation, while 1 week was selected as a short-term evaluation point to ensure early material maturation. On both sound and hypermineralized dentin, maturation was not directly visible in a significant change in bond strength or failure pattern of the tested materials but manifested itself in an increased reliability of the bond (Table 2, Weibull modulus m). In contrast, on artificially demineralized dentin, a significant drop was observed with aging along with visible changes in the fracture mechanisms. As the reduction was apparent for each material, the main factor seems to be the substrate’s ability to interact with the materials and its susceptibility to degradation. Insufficient development of the ion-exchange layer due to mineral depletion and increased exposure of the collagen structure may limit the ionic interactions and subsequent long-term adhesion [37]. A better understanding of the structural differences and preparation methods is important for further interpretation of the bonding performance on the altered substrates.

Laboratory models for altering the dentin were preferred in this study for the advantages of standardization, simplicity, affordability, and controlled reproducibility. Although it is not possible to completely mimic the clinical structure of altered dentin, in vitro models are capable of simulating chemical changes in a short period of time [38]. Rather than providing a direct equivalence to clinical dentin conditions, these artificially created substrates offer insight into material interactions with altered dentin under standardized conditions.

The artificial hypermineralization method used in this study was shown to be able to create a thin (app. 3 µm thick) surface layer of mineral deposits that did not penetrate into the dentinal tubules [39]. A reduction in the bond strength of various adhesive systems on hypermineralized dentin has been reported by Perdigao et al. [7], indicating that mineral deposits obliterating the tubules and intertubular dentin may hinder bonding. On the other hand, etch-and-rinse adhesive systems performed similarly on artificially hypermineralized dentin compared to sound dentin [39], possibly due to the removal of the surface layer by etching. Increased bond strength on the hypermineralized dentin when using methacryloyloxydecyl dihydrogen phosphate (MDP)-containing adhesives able to bond chemically was shown in a recent study [40]. The results of this study are in line with the present study, in which the MDP-containing primer used together with CF significantly improved the bond strength when compared to the sound dentin after one week of aging. Under extended aging conditions, the difference is less pronounced, but still noticeable at the limit of statistical significance (Table 2). Similarly, the chemical bonding of MDP-containing primer could have contributed to the higher reliability and consistent bonding of CF on hypermineralized dentin compared to sound dentin. Information on bonding to hypermineralized dentin is very limited for GICs in the literature. A recent study [28] reported a negative effect on high-viscosity GIC, while no deterioration was observed on the ion-releasing RBC and RMGIC.

The demineralized dentin was simulated by a pH-cycling method [22], which is the most relevant method due to similar mineral uptake and loss phases of the natural dynamic caries process found in the oral cavity [41]. Previous studies reported demineralization depths around 150 µm by 14-day pH cycling, albeit under varying pH conditions [42,43]. The SEM (Figure 4) and EDX (Figure 5) analysis of the demineralized dentin confirmed that the dentinal tubules were enlarged in comparison to the hypermineralized dentin surface, along with a demineralization depth of approximately 100 µm. As a previous study reported [7], the demineralized area was observed as a depression when dehydrated. This observation reflects the structural characteristics of dentin with insufficient inorganic components, high water content, and a degraded organic matrix. It must be considered that the accelerated caries formation methods do not completely mimic natural caries lesions and may differ among studies, leading to difficulties in comparison due to variations. Nevertheless, comparison and ranking of the materials help to make a general assumption. The reduced bonding performance of GICs and RMGICs on demineralized dentin was demonstrated in previous studies [32,44,45]; however, the extent of the destructive effect observed in the present study has not been commonly reported. Although CF showed the highest value on demineralized dentin after 1 week of storage, the reduction was steeper than that of EXP. The bond strength of EXP was reduced by half after 1 week, while the change was the most aggressive for FJLC. This led to GIC having a higher bond strength than RMGIC on demineralized dentin, in contrast to the literature [32,44,46].

Ion-releasing materials have the potential to increase the mineral density in artificial caries lesions [47,48]. GIC-sourced ions were shown to cross the interface into both natural [48] and artificial caries lesions [47]. However, delivery of minerals alone might not be sufficient for remineralization. According to Kim et al. [37], despite the diffusion of ions from GIC into the demineralized dentin, apatite deposition was not observed, failing to remineralize completely mineral-depleted dentin. It was previously suggested that pre-existing nucleation sites are necessary to facilitate ion exchange with dentin that leads to the remineralization starting from innermost demineralized areas by mineral growth with nucleation sites [49,50]. Formation of the ion-enriched layer in the interface involves release of phosphate and calcium ions from the tooth structure [34]. The use of an aggressive artificial demineralization method could be considered a limitation for the understanding of the material performance, as the remineralization process of natural and artificial caries lesions may differ [50]. The strongly demineralized dentin in the current study may have lacked sufficient mineral content, providing less opportunity for the chemical reaction and not being able to induce remineralization. As for CF (and Cention N), bioactive properties have been demonstrated [17,51], along with the formation of an apatite-like phase, by releasing high levels of fluoride and calcium [17]. High Ca release [52] and tighter marginal sealing capacity of this material were attributed to continuous ion release and pH-regulating potential [51], which is also promising for the inhibition of secondary caries formation. The results of the current study led to questions regarding the remineralizing potential of the tested material on strongly degraded dentin, although the bond strength of CF to demineralized dentine was higher compared to the other tested materials.

The interface of GICs and RMGICs on demineralized dentin is prone to hydrolytic degradation [46,53,54], as are the adhesive interfaces [55]. Water is a known degradation factor on the bonded interfaces. Both CF primer and FJLC contain HEMA, which is a frequent hydrophilic monomer found in many adhesive systems. HEMA promotes water sorption and leads to an unstable interface susceptible to hydrolytic degradation after prolonged water storage [56]. This causes plasticization of the HEMA-containing materials [57]. Moreover, water sorption of FJLC was shown to be higher than Cention N in a recent study [25]. Although a reduction in FJLC bond strength on artificial caries-affected dentin was previously reported [32,45], no study has reported complete failure of this material. In a recent study, a high number of pre-test failures (52.5%) for another RMGIC bonded to natural caries-affected dentin was also reported, along with a significant drop in bond strength after 1 year of storage and the presence of a gap between the material and dentin after aging [53]. The extreme reduction of the bond for CF, resulting in similar performance to EXP after 6 months, may be a result of the insufficient amount of mineral remaining in the demineralized dentin to interact with MDP, despite its known contribution to the stability of the interface over time [58]. Furthermore, application of cavity conditioner to the already mineral-depleted demineralized dentin surface may have removed a substantial portion of the limited remaining mineral content, leaving insufficient calcium available for adequate chemical bonding with FJLC. This could partly contribute to the poor bonding observed with FJLC in the present study, even though cavity conditioner is reported to be beneficial on sound dentin. [27] However, the conditioner’s individual effect cannot be isolated from that of FJLC itself, as it was applied only with this material. The results of FJLC are striking, as it performed the worst when bonded to demineralized dentin, with all specimens failing prior to testing after aging. The findings of the current study could suggest the high susceptibility of all tested materials to highly destructive conditions. Due to the reduced bonding performance on demineralized dentin and reduction over time, the second and third null hypotheses that dentin alteration and aging would not affect the bond strength are rejected. The clinical implications of these findings require cautious interpretation as the overall low bond strength values limit the validity of broad clinical extrapolations. Nevertheless, the complete failure of FJLC with cavity conditioner suggests its potential inadequacy when applied to severely degraded dentin. CF and EXP could be more favorable than FJLC in cases where bonding to mineral-depleted dentin is necessary. Clinical relevance of bonding to simulated demineralization remains to be further evaluated.

SBS testing is a commonly used method for evaluating the adhesion of brittle materials such as GICs. Even though bond strength testing does not provide direct clinical prediction, it allows material comparison under controlled conditions. Despite the limitation of non-uniform stress distribution and a larger bonding area with increased probability of a strength-limiting flaw compared to the microtensile bond strength test [59], SBS testing reduces the risk of damaging the brittle materials during cutting and trimming procedures. Mechanical properties (e.g., reduced elastic modulus, hardness) of both material and altered substrates [41,49] could affect the SBS of the materials. Demineralized dentin exhibits viscoelastic properties [60], showing elongation and stress absorption consistent with low modulus [61], as observed during the testing of demineralized dentin groups for each material. The mechanical properties of the GICs and RMGICs could be negatively influenced by the high water content of the demineralized substrate since they are susceptible to water uptake and loss during the initial setting reaction that affects the setting reaction [62]. After 1 week of storage, failures of EXP specimens were more catastrophic, with cohesive bulk fractures mostly on the edges of the material, unlike the cohesive failures in other groups. This observation leads to the question regarding the maturation of the material. Although the moisture susceptibility of RMGICs is less than that of GICs according to the literature [63], FJLC was influenced by the demineralized dentin the most, showing the lowest bond strength that failed completely adhesively.

The information gained from fractographic analysis is crucial when evaluating the bond strength of GIC-based materials due to differences in the literature. The probability of a critical flaw, such as microcracks or pores, is more common for GICs that affect the pattern of the fracture, especially on larger bonded surfaces. GICs and RMGICs are susceptible to cohesive failures, as they are both very brittle materials [64]. Very frequently in the literature, fractures that appeared like a peeled-off layer on the dentin surface, often close to the interface, were also considered cohesive fractures [44,64]. In the current study, only specimens exhibiting bulk fracture of dentin or material were classified as cohesive failures. These types of fractures do not represent the interface bond strength, but a mixture of mechanical properties of different structures [59]. Since the surface area involved in the fracture of cohesive failures is larger than the bonded area, the bond strength cannot be calculated correctly. In the current study, adhesively fractured specimens were further examined according to the remaining thin layer of material on the dentin surface. Remnants were observed on FJLC and EXP specimens, possibly due to the interaction layer, as it was not common for the CF specimens. The number of specimens with material remnant covering more than 75% of the surface was higher for EXP. Similar to our findings, failure above the hybrid layer, with the material remnant covering the debonded interface, was reported for FJLC previously [53,54].

Fractographic analysis revealed a distinctive interfacial structure at the demineralized dentin surface. A thin layer was observed between the restorative materials and the demineralized dentin, which was not visible on sound and hypermineralized dentin surfaces. This layer’s tendency to shrink upon drying, together with its loose attachment, suggests that the moist structure of demineralized dentin may have contributed to its formation. A non-particulate, structureless “absorption layer” has been previously reported between RMGIC and sound, hydrated deep dentin and was associated with water movement [65,66]. However, while that layer was previously assumed to mediate improved bond strength, the layer identified in the present study was specific to demineralized dentin and was associated with markedly reduced bond strength. Further chemical and ultrastructural analysis of this observed layer could help clarify its formation.

Weibull analysis provides relevant information on the reliability of the bond and failure probability. A lower Weibull modulus reflects higher variability in the bond strength, which means lower reliability of the bond strength due to critical flaws [67]. CF exhibited less scatter and greater consistency in bond strength, reflecting a steeper Weibull failure probability curve. This indicates more predictable clinical performance than FJLC and EXP. Although the aging effect on bond strength was not explicit, the reliability of CF and EXP increased on sound dentin, while a slight reduction was observed for FJLC. Even though EXP experienced an increase in Weibull modulus after aging on sound dentin, the opposite was observed on hypermineralized dentin. The combination of higher reliability and higher SBS suggests that CF may be a more suitable material than FJLC and EXP when bonding to sound or sclerotic dentin.

## 5. Conclusions

This study has shown that material type and substrate properties exert a strong and comparable influence on the SBS value, whereas aging plays a minor role. Ion-releasing RBC has superior bonding performance and reliability on sound and hypermineralized dentin compared to RMGIC and GIC. Artificially demineralized dentin exhibited a severely destructive impact, leading to very low bond strength of all materials. The integrity of the adhesive interface could not be preserved following maturation on demineralized dentin, unlike on sound dentin.

## Figures and Tables

**Table 1:** Chemical composition of the materials used.

Commercial Name (Abbreviation)	Type of Material	Composition	LOT Number
Cention Forte (CF)	Ion-releasing RBC	UDMA, calcium fluoro-silicate glass, aromatic-aliphatic UDMA, copolymer, barium-aluminum silicate glass, calcium-barium-aluminum fluoro-silicate glass, DCP, PEG-DMA, barium glass, and ytterbium trifluoride.	ZL08SV
Cention Primer		HEMA, Bis-GMA, MDP, ethanol, water, D3MA, methacrylate-modified polyacrylic acid, silicon dioxide and campherquinone	Z03F0P
Fuji II LC Capsule (FJLC)	RMGIC	Powder: Fluoro-alumino-silicate glass, initiator, pigment. Liquid: HEMA, UDMA, polyacrylic acid, dimethacrylate, carboxylic acid, water, initiator, stabilizer.	221222A
Cavity Conditioner		Polyacrylic acid, aluminum chloride hexahydrate	2301211
GC Fuji Varnish		Acetone, isopropyl acetate, cornmint oil, cinnamaldehyde	2210211
Experimental GI Filling Material (EXP)	GIC	Glass, acrylic acid copolymer, water, tartaric acid	Exp

Abbreviations: UDMA—urethane dimethacrylate; DCP—tricyclodecane dimethanol dimethacrylate; PEG-DMA—polyethylene glycol dimethacrylate; HEMA—2-hydroxyethyl methacrylate; Bis-GMA—bisphenol A-Glycidyl methacrylate; MDP—methacryloyloxydecyl dihydrogen phosphate; D3MA—1,10-decanediol dimethacrylate; RBC—resin-based composite; GIC—glass ionomer cement; RMGIC—resin-modified GIC.

**Table 2:** Shear bond strength (SBS) in MPa (mean ± standard deviation), pre-test failures (ptfs) per number of specimens of the group, Weibull modulus (m) with 95% confidence interval in brackets and R^2^ values. Different uppercase letters in each row and lowercase letters in each column within one aging category indicate significant differences in SBS between materials. Asterisk (*) indicates significant influence of aging. Material abbreviations can be found in Table 1.

		Sound		Hypermineralized		Demineralized
		SBS (ptf/n)	m R^2^	SBS (ptf/n)	m R^2^	SBS (ptf/n)
**1 week**	CF	19.7 ± 7.7 ^Ba^ (0/20)	2.6 (2.4; 2.9) 0.95	26.8 ± 6.3 ^Aa^ (0/20)	4.9 (4.1; 5.6) 0.92	5.3 ± 3.4 ^Ca^ (0/20)
	FJLC	8.8 ± 4.3 ^Ab^ (0/20)	1.6 (1.4; 1.8) 0.93	9.9 ± 4.5 ^Ab^ (0/20)	1.5 (1.2; 1.8) 0.87	0.8 ± 0.8 ^Bc^ (4/20)
	EXP	4.9 ± 2.8 ^Ac^ (0/20)	1.8 (1.6; 1.9) 0.96	4.6 ± 2.1 ^Ac^ (0/20)	2.2 (2.0; 2.4) 0.96	2.5 ± 1.4 ^Bb^ (0/20)
**6 months**	CF	20.6 ± 6.1 ^Aa^ (0/20)	3.8 (3.5; 4.1) 0.98	23.8 ± 6.3 ^Aa^ (0/20)	4.5 (4.0; 5.0) 0.95	0.5 ± 0.3 ^Ba^* (2/20)
	FJLC	7.3 ± 4.9 ^Ab^ (0/20)	1.1 (1.0; 1.3) 0.94	9.4 ± 5.6 ^Ab^ (0/20)	1.3 (1.2; 1.4) 0.96	0 ^Bb^* (20/20)
	EXP	4.8 ± 2.3 ^Ab^ (0/20)	2.3 (2.1; 2.6) 0.95	4.4 ± 2.8 ^Ac^ (1/20)	0.9 (0.7; 1.1) 0.85	0.3 ± 0.3 ^Ba^* (8/20)

**Table 3:** Parameters of the model used to fit the phosphorus (P) and calcium (Ca) variation data in the EDX line-scan profiles. a and b are presented with their 95% confidence intervals in brackets together with R^2^ for each of the three demineralized dentin specimens. Three measurements were performed in each specimen.

Mineral	Specimen	a	b	R^2^
**P**	P1	41.3 (40.6; 42.1)	0.024 (0.021; 0.027)	0.73
	P2	46.6 (45.4; 47.8)	0.013 (0.011; 0.014)	0.72
	P3	42.2 (41.2; 43.1)	0.017 (0.015; 0.020)	0.66
**Ca**	P1	34.2 (33.6; 34.9)	0.022 (0.019; 0.025)	0.69
	P2	34.8 (34.0; 35.6)	0.015 (0.013; 0.016)	0.72
	P3	39.7 (38.6; 40.7)	0.011 (0.010; 0.013)	0.75

## Data Availability

The raw data supporting the conclusions of this article will be made available by the authors on request.
